# Neonatal testicular torsion: a single-center 14-year experience in diagnosis and management

**DOI:** 10.3389/fped.2026.1749065

**Published:** 2026-04-14

**Authors:** Yi Sun, Xingquan Tu, Zhongxu Wang, Xiangming Yan, Yun Zhou, Mingcui Fu, Shu Dai, Qianwei Xiong, Hongliang Xia, Xu Cao, Ting Zhang, Xiyang Wang

**Affiliations:** Department of Urology, Children’s Hospital of Soochow University, Suzhou, Jiangsu, China

**Keywords:** neonate, orchidopexy, orchiectomy, testicular torsion, testis

## Abstract

**Objective:**

To delineate the clinical characteristics, diagnostic approaches, and therapeutic strategies for neonatal testicular torsion (NTT), while synthesizing a single-center 14-year management experience to refine protocols for early recognition and intervention.

**Methods:**

A retrospective analysis was conducted on 31 neonates with NTT managed in the Department of Pediatric Urology at the Children's Hospital of Soochow University between October 2010 and October 2024. Clinical data encompassed birth weight, body length, age at diagnosis, mode of delivery, presenting symptoms, preoperative ultrasonography, intraoperative findings, and follow-up outcomes. All patients underwent emergent surgical exploration, with orchidopexy or orchiectomy performed based on intraoperative assessment of testicular viability; contralateral prophylactic orchidopexy was undertaken in select cases. Statistical analysis was performed using SPSS version 21.0. Continuous variables were expressed as mean ± standard deviation or median (interquartile range), and categorical variables as frequencies (percentages).

**Results:**

In this retrospective series of 31 neonates with NTT, unilateral involvement occurred in 29 cases and bilateral in 2 (6.45%). In the 29 neonates with unilateral NTT, the mean birth weight was 3.33 ± 0.65 kg, with a median age at diagnosis of 3.00 days (IQR: 1.00–10.00). Left-sided torsion predominated (62.07%, 18/29). Predominant manifestations included scrotal erythema (82.76%, 24/29) and induration (62.07%, 18/29). Preoperative color Doppler ultrasonography revealed absent intratesticular blood flow in 96.55% (28/29) of affected testes. Intraoperatively, 96.55% (28/29) of testes were nonviable and necessitated orchiectomy, yielding a salvage rate of only 3.45% (1/29). The median degree of torsion was 630° (IQR: 360.00°–720.00°), with extravaginal torsion accounting for 75.86% (22/29). Contralateral prophylactic orchidopexy was completed in 79.31% (23/29) of cases. Over a median follow-up of 72 months, no contralateral torsion was observed. In the bilateral cases, Case 1 was asynchronous bilateral torsion: the left testis showed 360° torsion with ischemic necrosis requiring orchiectomy, while the right testis had viable perfusion after detorsion and was preserved with orchidopexy; follow-up showed no atrophy and good blood supply. Case 2 was synchronous bilateral torsion with obvious bilateral ischemia and necrosis intraoperatively; bilateral orchidopexy was performed to preserve potential Leydig cell function, but follow-up revealed bilateral testicular atrophy.

**Conclusion:**

NTT predominantly manifests as unilateral, extravaginal torsion, posing challenges to early detection and resulting in low testicular salvage rates. Color Doppler ultrasonography emerges as a pivotal diagnostic modality. Emergent surgical exploration coupled with contralateral prophylactic orchidopexy may help reduce the risk of complications. This study provides data supporting prompt diagnosis and surgery in NTT.

## 1 Introduction

NTT represents a rare urological emergency, with an incidence of approximately 6.1 per 100,000 live births, accounting for 10%–12% of all pediatric testicular torsion cases (1–3). NTT is classified as intrauterine (approximately 70%–80%) or postnatal (occurring within 30 days of birth) based on timing of onset (4, 5). The condition arises from abnormal axial rotation of the testis and spermatic cord, resulting in vascular compromise; without prompt intervention, ischemic necrosis ensues, potentially compromising reproductive function (3). NTT is predominantly unilateral, with bilateral involvement being substantially less common (6). Bilateral cases are further categorized as synchronous or asynchronous based on temporal sequence (7). Because bilateral torsion can cause severe and irreversible damage, clinicians should remain alert to this possibility.

Recent studies on NTT have focused on its etiology, diagnosis, and treatment. Some evidence suggests that perinatal stress and an active cremasteric reflex may play a role in NTT (8). Complicated pregnancies, such as twins, maternal diabetes, and macrosomia, have been linked to higher risk (4, 9, 10). Additionally, contralateral hydrocele has been increasingly recognized as a potential risk factor for NTT in recent years (1, 11). Color Doppler ultrasonography has emerged as the diagnostic cornerstone, demonstrating sensitivity of 89.9% and specificity of 98.8% (12). Therapeutic controversy persists regarding the necessity of emergent surgical exploration. Detorsion with orchidopexy or orchiectomy constitutes the primary operative approaches, with procedural selection depending on torsion degree and tissue viability. Many studies show very low salvage rates for intrauterine NTT, but postnatal cases treated within 6 h of symptom onset have much better testicular survival (13).

Despite diagnostic advances, early recognition of NTT remains difficult, as neonates cannot express pain and are often misdiagnosed with torsion of the testicular appendage or orchitis. Intrauterine NTT typically presents with irreversible parenchymal damage at birth, so timely treatment has limited effect. The lack of standardized multicenter protocols increases the risk of misdiagnosis and treatment delay, leading to testicular loss and other complications. This study aims to improve diagnostic and treatment approaches and highlights the need for early detection and surgical intervention in NTT management.

## 2 Patients and methods

This retrospective review included 31 neonates with NTT treated at our institution between October 2010 and October 2025, accounting for 33 involved testes (including two cases of bilateral NTT). Inclusion criteria comprised: ① postnatal age <30 days; ② intraoperative confirmation of testicular torsion; ③ complete clinical records with a minimum follow-up of 12 months. Exclusion criteria were: ① postnatal age ≥30 days; ② concomitant cryptorchidism, incarcerated hernia, or other confounding pathologies; ③ incomplete clinical documentation or absent follow-up data. The study protocol was approved by the Institutional Review Board of the Children's Hospital of Soochow University (Ethics No. 2025CS165), with waiver of informed consent.

## 3 Research methods

**Figure 1:**
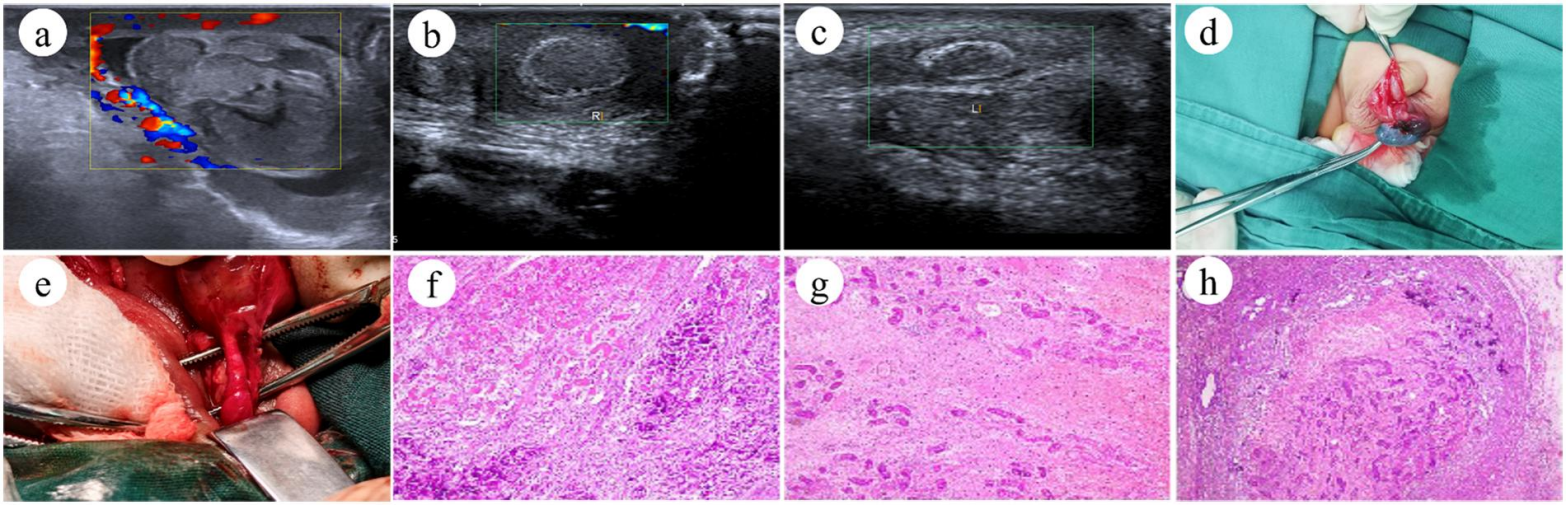
Preoperative ultrasonography, intraoperative findings, and postoperative histopathology of NTT. **(a)** The left testis measures 13 mm × 8 mm with altered axial orientation; heterogeneous echotexture is observed in both the testis and epididymis, accompanied by capsular thickening (2.2 mm) with irregular echogenicity. Color Doppler flow imaging (CDFI) demonstrates absent blood flow signals in the left testis and epididymis. **(b,c)** Bilateral testicular contour deformity is evident, with right-sided enlargement and left-sided atrophy. The left testis measures 7 × 4 mm with enhanced, heterogeneous echotexture; the right measures 10 × 6 mm with relatively homogeneous internal echoes. Eggshell-like calcifications are present bilaterally. CDFI reveals no intratesticular or subcapsular blood flow signals on either side. **(d)** Intraoperative exposure of the affected testis reveals a blackened appearance. **(e)** Extravaginal torsion, with torsion direction and degree no longer discernible. **(f–h)** Histopathology demonstrates focal hemorrhagic necrosis, scattered hemosiderin deposition, surrounding fibroblastic proliferation, and focal calcification.

Data collection primarily encompassed the following parameters: age at onset, body weight, body length, comorbidities, mode of delivery, prematurity status, laterality of testicular torsion, clinical manifestations, preoperative color Doppler ultrasonography demonstrating presence or absence of blood flow in the affected testis (Figures 1a–c), surgical intervention on the ipsilateral testis, and prophylactic fixation of the contralateral testis. Intraoperative findings included direction and degree of torsion, restoration of testicular perfusion following detorsion, intravaginal vs. extravaginal torsion, operative duration, intraoperative blood loss, contralateral torsion or developmental anomalies during follow-up, and follow-up duration. Successful testicular salvage was defined as the absence of atrophy on long-term follow-up physical examination and color Doppler ultrasonography, with preserved good blood flow signals. Details of the bilateral NTT cases are listed separately in Tables 1, 2. Color Doppler ultrasonography was performed by ultrasound physicians using a Canon TUS-A400 or Philips IU ELITE ultrasound system, with a 10–14 MHz linear probe. The neonate was placed in the supine position with the scrotum fully exposed. Coupling gel was applied to the scrotal surface, and the probe was placed directly on the scrotum. Scanning was conducted by sliding the probe over both sides and the superior-posterior aspect of the scrotum, including transverse, longitudinal, and bilateral comparative views. The size, morphology, echogenicity, and blood flow signals of the testis, epididymis, and testicular appendages were evaluated, along with blood flow in the spermatic cord and the presence or absence of fluid collection in the tunica vaginalis cavity.

**Table 1:** Baseline data of neonatal testicular torsion.

Variables	Unilateral NTT	Bilateral NTT Case 1		Bilateral NTT Case 2	
	(*n* = 29)	Left	Right	Left	Right
Birth weight (kg), Mean ± SD	3.33 ± 0.65	4		3.05	
Body length (cm), M (Q_1_, Q_3_)	50.00 (49.00, 51.00)	55		46	
Mode of delivery, *n* (%)					
Vaginal delivery	13 (44.83)	Yes		no	
Cesarean section	16 (55.17)	no		yes	
Prematurity, *n* (%)					
no	26 (89.66)	no		no	
yes	3 (10.34)				
Laterality, *n* (%)					
Left	18 (62.07)	–	–	–	–
Right	11 (37.93)	–	–	–	–
Pathogenetic process					
Age at diagnosis (days), M (Q_1_, Q_3_)	3.00 (1.00, 10.00)	18	18	0	0
Age at diagnosis classification					
≤24hours	7 (24.14)	no		yes	
24 h—7 days	14 (48.28)	no		no	
>7 days	8 (27.59)	yes		no	
Duration of symptoms^a^ (hours), M (Q_1_, Q_3_)	23.00 (8.00, 48.00)	Indeterminate	Indeterminate	3	3
Clinical manifestations, *n* (%)					
Scrotal erythema	24 (82.76)	yes	yes	no	no
Scrotal induration	18 (62.07)	yes	yes	yes	yes
Tenderness	9 (31.03)	no	yes	no	no
Fever	3 (10.34)	yes	no		
Ipsilateral testicular enlargement	4 (13.79)	no	no	no	no
Asymptomatic, detected by ultrasound	1 (3.45)	no	no	no	no
Concomitant hydrocele, *n* (%)	19 (65.52)	no	yes	no	no

SD, standard deviation; M, Median; Q_1_, 1st Quartile; Q_3_, 3rd Quartile.

^a^ except for 1 asymptomatic case.

**Table 2:** Preoperative, intraoperative, and follow-up conditions.

Variables	Unilateral NTT	Bilateral NTT Case 1		Bilateral NTT Case 2	
	(*n* = 29)	Left	Right	Left	Right
Preoperative blood flow in affected testis, *n* (%)					
Absent	28 (96.55)	yes	no	yes	yes
Present	1 (3.45)	no	yes	no	no
Ipsilateral testicular management, *n* (%)					
Orchidopexy	1 (3.45)	no	yes	yes	yes
Orchiectomy	28 (96.55)	yes	no	no	no
Contralateral orchidopexy	23 (79.31)	-	-	-	-
Ipsilateral testicular salvage, *n* (%)	1 (3.45)	no	yes	no	no
Direction of torsion, *n* (%)					
Clockwise	5 (17.24)	no	yes	no	no
Counterclockwise	13 (44.83)	yes	no	no	yes
Indeterminate	11 (37.93)	no	no	yes	no
Intraoperative testicular color, *n* (%)					
Black	28 (96.55)	yes	no	yes	yes
Restored	1 (3.45)	no	yes	no	no
Site of torsion, *n* (%)					
Extravaginal	22 (75.86)	yes	yes	no	yes
Intravaginal	5 (17.24)	no	no	no	no
Indeterminate	2 (6.90)	no	no	yes	no
Degree of torsion (degrees), M (Q_1_, Q_3_)	630.00 (360.00, 720.00)	360	90	Indeterminate	720
Operative duration (min), M (Q_1_, Q_3_)	39.00 (35.00, 45.00)	40		82	
Intraoperative blood loss (mL), M (Q_1_, Q_3_)	2.00 (1.00, 3.00)	1		2	
Follow-up duration (months), M (Q_1_, Q_3_)	72.00 (36.00, 96.00)	15		12	

SD, standard deviation; M, Median; Q_1_, 1st Quartile; Q_3_, 3rd Quartile.

### 3.1 Surgical techniques

All patients underwent emergent surgical exploration under combined inhalational and intravenous general anesthesia. The neonate was positioned supine, followed by standard skin preparation and draping. A 2-cm transverse scrotal incision was made on the affected side, sequentially incising the skin, subcutaneous tissue, dartos fascia, and tunica vaginalis to expose the testis (Figure 1d). Testicular color and perfusion were meticulously assessed. Torsion type and degree were identified (Figure 1e), followed by immediate detorsion. The testis was then wrapped in warm saline-soaked gauze for a minimum of 15 min to evaluate perfusion recovery. If robust revascularization was observed, orchidopexy was performed. In cases of persistent ischemia with dark-purple discoloration, a stab incision was made through the tunica albuginea into the medullary parenchyma to assess arterial bleeding latency, graded according to the Arda three-tier system (14) (Grade I: immediate arterial oozing upon incision, indicating viable parenchyma—orchidopexy proceeded; Grade II: arterial bleeding within 10 min—testis preserved with fixation; Grade III: no bleeding at 10 min, signifying irreversible necrosis—orchiectomy was performed with parental consent). Contralateral prophylactic orchidopexy was undertaken in some patients, and all resected specimens underwent histopathological examination (Figures 1f–h). Since 2014, bilateral exploration with routine fixation of the contralateral testis has been adopted as our institutional standard protocol to allow simultaneous evaluation of the affected gonad and prevention of metachronous torsion in the contralateral testis, thereby markedly reducing the risk of anorchia.

### 3.2 Statistical analysis

Statistical analyses were performed using SPSS version 21.0. Normality of continuous variables was assessed with the Shapiro–Wilk test. Normally distributed data are expressed as mean ± standard deviation, whereas skewed data are presented as median (interquartile range, Q1–Q3). Categorical variables are reported as frequencies (percentages).

## 4 Results

### 4.1 General information

This retrospective study analyzed the clinical profiles of 31 neonates with NTT. Of these, 29 presented with unilateral NTT, while 2 had bilateral involvement (with the bilateral cases presented separately in the table). As shown in Table 1, among the 29 neonates with unilateral NTT, the mean birth weight was 3.33 ± 0.65 kg, with a median body length of 50.00 cm (IQR: 49.00–51.00 cm) and a median age at diagnosis of 3.00 days (IQR: 1.00–10.00 days). Delivery was by cesarean section in 55.17% (16/29) and vaginal in 44.83% (13/29). Prematurity accounted for 10.34% (3/29). Torsion laterality was predominantly left-sided (62.07%, 18/29), followed by right-sided (37.93%, 11/29). Principal clinical manifestations encompassed scrotal erythema (82.76%, 24/29), induration (62.07%, 18/29), tenderness (31.03%, 9/29), fever (10.34%, 3/29), and ipsilateral testicular enlargement (13.79%, 4/29). Concomitant hydrocele was observed in 65.52% (19/29).

### 4.2 Intraoperative and postoperative outcomes

Table 2 delineates intraoperative and follow-up parameters. In the 29 neonates with unilateral NTT, preoperative color Doppler ultrasonography revealed absent blood flow in 96.55% (28/29) of affected testes. During surgical exploration, orchiectomy was necessitated due to necrosis in 96.55% (28/29) of cases, with orchidopexy performed in only 3.45% (1/29); the ipsilateral testicular salvage rate was a mere 3.45% (1/29). Intraoperatively, 96.55% (28/29) of testes exhibited a blackened hue, with perfusion restored to normal coloration in just 3.45% (1/29). Among the 29 unilateral torsion events, the plane of twist could not be determined in 2 cases (6.90%). Extravaginal torsion was the predominant mechanism (75.86%, 22/29), while intravaginal torsion occurred in 5 cases (17.24%). Torsion degree was quantifiable in 18 units, with a median of 630° (IQR: 360.00–720.00°). Notably, one neonate was admitted for hypoglycemia and pneumonia without any obvious signs of testicular torsion. Routine scrotal ultrasound incidentally detected left-sided NTT. Intraoperative findings showed 180° torsion with good restoration of blood supply after detorsion, allowing preservation of the testis. In bilateral torsion Case 1, the right testis showed 90° torsion and restored adequate blood flow after detorsion, allowing preservation. These salvaged testes exhibited lower degrees of torsion. Counterclockwise rotation accounted for 44.83% (13/29) of unilateral cases, clockwise rotation for 17.24% (5/29), while the direction of torsion could not be determined in the remaining 37.93% (11/29). Contralateral prophylactic orchidopexy was completed in 79.31% (23/29). Median operative duration was 39.00 min (IQR: 35.00–45.00 min), with median intraoperative blood loss of 2.00 mL (IQR: 1.00–3.00 mL). Median follow-up spanned 72.00 months (IQR: 36.00–96.00 months), during which no contralateral torsion occurred; the contralateral testes showed no atrophy and demonstrated normal blood flow signals on color Doppler ultrasonography. The two salvaged testes showed good blood flow on long-term postoperative follow-up. In unilateral torsion cases, long-term follow-up color Doppler ultrasonography demonstrated that the volume of the affected testis was greater than 50% of the contralateral testis. In bilateral NTT Case 1, long-term follow-up of the right testis revealed good blood flow, normal tissue density, and normal testicular development.

### 4.3 Bilateral NTT

Case 1: Bilateral scrotal swelling was noted at birth and diagnosed as hydrocele at the local hospital. At 18 days, ultrasound at our center suggested bilateral extravaginal torsion. Emergent surgery revealed counterclockwise 360° extravaginal torsion of the left testis with dark-purple discoloration and poor blood supply after detorsion; Arda test showed no arterial oozing, leading to left orchiectomy. The right testis had clockwise 90° extravaginal torsion with adequate blood supply after detorsion, allowing preservation. Based on intraoperative ischemia severity, this was asynchronous bilateral torsion. Long-term follow-up ultrasound showed good blood flow, normal tissue density, and normal development of the preserved right testis.

Case 2: At birth, the obstetrician noted slightly dark and reddish bilateral scrotal color and recommended transfer to our hospital. Emergency ultrasound indicated bilateral testicular torsion. Emergent exploration revealed counterclockwise 720° extravaginal torsion of the right testis with poor blood flow recovery after detorsion and no arterial oozing on Arda test. The left testis showed adhesions and fibrosis to the tunica vaginalis sac, making torsion site, direction, and degree unrecognizable. Considering potential Leydig cell function, bilateral orchidopexy was performed with parental consent. This was synchronous bilateral torsion. Long-term follow-up showed bilateral testicular atrophy.

## 5 Discussion

This retrospective review of 31 neonates with NTT describes the main clinical features and treatment difficulties. In the 29 cases of unilateral NTT, extravaginal torsion accounted for 75.86% (22/29), with left-sided involvement predominating in 62.07% (18/29) and a median torsion degree of 630°. Preoperative color Doppler ultrasonography demonstrated absent perfusion in 96.55% (28/29) of affected testes, indicative of pervasive ischemic injury, culminating in orchiectomy for necrosis in 96.55% (28/29) of cases and a salvage rate of only 3.45% (1/29). Principal manifestations included scrotal erythema (82.76%, 24/29) and induration (62.07%, 18/29); however, nonspecific symptoms in neonates often led to misdiagnosis. Contralateral prophylactic orchidopexy was performed in 79.31% (23/29) of unilateral patients, with a median follow-up of 72 months revealing no contralateral torsion events. These findings show the difficulty of early diagnosis and the need for emergent surgical intervention.

The etiology of NTT remains incompletely understood and likely involves a complex interplay of multiple factors related to the neonate and maternal gestation. Neonatal factors include poor adhesion of the tunica vaginalis to the scrotal wall, predisposing to extravaginal torsion [seen in 75.86% (22/29) of unilateral cases in our series], elevated attachment of the parietal tunica along the spermatic cord, and an elongated, loose gubernaculum resulting in the “bell-clapper” deformity. An active cremasteric reflex in neonates may also increase the risk of torsion (8, 15). Moreover, neonatal testicular tissue has low tolerance to ischemia, which can worsen outcomes (16). Notably, our cohort showed a high concomitant hydrocele rate. In one bilateral NTT case (case 1), the neonate presented to local hospital shortly after birth with bilateral scrotal swelling diagnosed as bilateral hydrocele; at 18 days of age, admission to our center confirmed bilateral testicular torsion with associated right hydrocele. This highlights how hydrocele may mask NTT signs, complicating early diagnosis. Similarly, Omran et al. reported a case of NTT incidentally detected in a neonate with bilateral hydrocele, emphasizing the frequent association between NTT and hydrocele, the resultant diagnostic challenges, and treatment complexities (11). Maternal gestational factors implicated include gestational diabetes, large-for-gestational-age infants, breech presentation, prematurity, and protracted vaginal delivery. Kaye et al. found that 50% of cases had such risk factors (15), while a meta-analysis by Monteilh et al. of 196 NTT cases showed higher rates after vaginal delivery, possibly due to mechanical stress during birth (17). In our series, one successfully salvaged testis was incidentally detected on admission ultrasonography in a neonate hospitalized for hypoglycemia and pneumonia, absent overt torsion signs; intraoperative detorsion of 180° yielded viability. Maternal history disclosed gestational hyperglycemia, macrosomia, and obstructed vaginal delivery. Nonetheless, vaginal delivery constituted only 45.16% (14/31) of our cohort, potentially reflecting limited sample size. Because our center is a specialized children's hospital, relevant risk factors for NTT during pregnancy and the perinatal period were not consistently documented. As a result, this information is lacking in our study. In the future, we will prospectively collect such data to investigate pregnancy- and perinatal-related risk factors for NTT and thereby optimize the management of affected neonates.

Clinical presentation coupled with ultrasonography constitutes the cornerstone of NTT diagnosis; however, the nonspecific symptomatology in neonates frequently precipitates missed diagnoses. Neonates exhibit diminished pain sensitivity and inability to express discomfort, often leading to misdiagnosis as torsion of the testicular appendage or scrotal hematoma. Compounded by the exquisite ischemic vulnerability of neonatal testicular parenchyma, necrosis rates are exorbitant, with salvage achievable in only approximately 5% of cases (18, 19)—our institutional rate of 6.06% (2/33) aligns closely with prior reports. Ultrasonography is the primary imaging tool, showing characteristic features such as the “whirlpool sign”, absent blood flow, testicular enlargement, and capsular thickening (20). In our unilateral NTT cases, preoperative ultrasonography demonstrated absent intratesticular flow in 96.55% of cases, mirroring the intraoperative necrosis prevalence. However, due to the retrospective design and the inclusion of only surgically confirmed torsion cases, sensitivity and specificity could not be formally calculated in this study. Further prospective research is required to more definitively establish the diagnostic performance of color Doppler ultrasonography in NTT.

Current controversies in NTT management encompass surgical vs. conservative approaches, with the latter rooted in the traditional view of predominantly intrauterine torsion and low salvage rates (21); our center favors emergent surgery to mitigate risks of scrotal infection, testicular atrophy, long-term contralateral impairment, and potential oncogenesis—one 18-day-old neonate in our series presented with intraoperative scrotal abscess, underscoring the value of early intervention to avert complications. The necessity of bilateral exploration and contralateral orchidopexy has also been debated; since 2014, our protocol has incorporated contralateral fixation to prevent asynchronous torsion, enabling simultaneous ipsilateral viability assessment and contralateral securing, which may reduce the risk of anorchia—Ahmed et al. (22) identified intraoperative bilateral torsion in two cases despite unilateral preoperative swelling and a normal contralateral appearance (22), while Monteilh et al.'s meta-analysis (17) showed that bilateral exploration can salvage about 7% of affected testes and prevent 4% of asynchronous torsion events, with 8%–12% of patients potentially benefiting from bilateral surgery. This supports the value of bilateral exploration. Management of contralateral hydrocele is also controversial. Its incidence is high in NTT [approaching 60% in prior studies (1) and 65.52% (19/29) in our unilateral NTT cases, vs. 1%–5% in general neonates (23)], likely attributable to inflammatory exudation; considering the spontaneous resolution of hydroceles within 2 years (24, 25) and the risk of vasal or vascular injury from processus vaginalis ligation, our center employs expectant observation with close follow-up, deeming proactive intervention to forestall late indirect inguinal hernia unnecessary.

The strengths of this study include the detailed review of a single-center 14-year experience with 31 cases of NTT, providing data on clinical features, diagnostic methods, and treatment results. We recorded preoperative ultrasound, intraoperative findings, and long-term follow-up, which helped describe the typical unilateral extravaginal torsion pattern and the low salvage rates, while demonstrating the importance of emergent surgical exploration. Furthermore, a 79.31% completion rate for contralateral prophylactic orchidopexy in unilateral NTT cases coupled with no contralateral torsion events over a 72-month median follow-up underscores the clinical merit of bilateral exploration. Nonetheless, limitations include the modest sample size (*n* = 31), which may constrain generalizability; the single-center retrospective design introduces potential selection bias and fails to capture multicenter practice heterogeneity; and the inability to reliably stratify cases by torsion onset timing (prenatal vs. postnatal). The latter stems from limited access to prenatal records in our pediatric-only setting, frequent prenatal occurrence with occult presentation at birth, and postnatal delays often attributable to parental oversight or delayed recognition, which in turn introduce recall bias in symptom duration—despite the categorization of diagnosis timing and symptom duration in Table 1. These factors limit our ability to gain reliable insights into timing-dependent salvage rates. An additional limitation is that our study did not collect long-term endocrine and fertility outcomes. Future studies should prioritize multicenter prospective cohorts with detailed documentation of risk factors and symptom duration in NTT patients, as well as systematic collection of long-term endocrine and fertility outcomes to explore factors influencing reproductive prognosis. These cohorts should incorporate advanced imaging techniques and novel perfusion assessment tools, such as indocyanine green angiography (26), to refine early diagnostic algorithms, better evaluate long-term prognosis, and ultimately improve outcomes for NTT.

## 6 Conclusion

This study summarizes a single-center 14-year experience in managing NTT. Most cases were unilateral extravaginal torsion, with preoperative ultrasound showing no blood flow in 93.94% (31/33) of affected testes and a salvage rate of 6.06% (2/33). These results highlight the difficulty of early diagnosis. Emergent surgical exploration is essential for diagnosis and treatment. Prompt recognition, fewer misdiagnoses, and faster intervention can help improve testicular survival.

## Data Availability

The original contributions presented in the study are included in the article/Supplementary Material, further inquiries can be directed to the corresponding author/s.
